# VirulentPred 2.0: An improved method for prediction of virulent proteins in bacterial pathogens

**DOI:** 10.1002/pro.4808

**Published:** 2023-12-01

**Authors:** Arun Sharma, Aarti Garg, Jayashree Ramana, Dinesh Gupta

**Affiliations:** ^1^ Translational Bioinformatics Group International Centre for Genetic Engineering and Biotechnology (ICGEB) New Delhi India

**Keywords:** AutoGluon, bacterial proteins, classification, machine learning, prediction, virulence factors, virulent proteins, VirulentPred 2.0

## Abstract

Virulence proteins in pathogens are essential for causing disease in a host. They enable the pathogen to invade, survive and multiply within the host, thus enhancing its potential to cause disease while also causing evasion of host defense mechanisms. Identifying these factors, especially potential vaccine candidates or drug targets, is critical for vaccine or drug development research. In this context, we present an improved version of VirulentPred 1.0 for rapidly identifying virulent proteins. The VirulentPred 2.0 is based on training machine learning models with experimentally validated virulent protein sequences. VirulentPred 2.0 achieved 84.71% accuracy with the validation dataset and 85.18% on an independent test dataset. The models are trained and evaluated with the latest sequence datasets of virulent proteins, which are three times greater in number than the proteins used in the earlier version of VirulentPred. Moreover, a significant improvement of 11% in the prediction accuracy over the earlier version is achieved with the best position‐specific scoring matrix (PSSM)‐based model for the latest test dataset. VirulentPred 2.0 is available as a user‐friendly web interface at https://bioinfo.icgeb.res.in/virulent2/ and a standalone application suitable for bulk predictions. With higher efficiency and availability as a standalone tool, VirulentPred 2.0 holds immense potential for high throughput yet efficient identification of virulent proteins in bacterial pathogens.

## 1 INTRODUCTION

In recent decades, there has been a huge spike in infectious diseases caused by different pathogens, such as bacteria. Due to this, there is a strong need to find new antimicrobial therapies and drug targets for treatment. Although several drug targets are known, however due to the problem of antibiotic resistance (Ayukekbong et al., 2017) there is an ever‐increasing need to discover new targets for developing new drugs. Furthermore, with the availability of the complete sequence annotations of several pathogen genomes (Land et al., 2015), the protein sequences of several virulent factors have also become easily accessible.

Virulent proteins assist the bacterium to colonize the host at the cellular level, promoting pathogenicity (Denzer et al., 2020). In addition, the virulent factors interact with the host's immune system and are essential for pathogens to establish infection (Sarowska et al., 2019), thus contributing directly and indirectly to disease processes. The virulent factors are classified into different types based on their crucial role during the disease processes such as adhesion (Patel et al., 2017), invasion, colonization, toxins (Foster et al., 2014), and so forth. All virulence factors employed by pathogens and bacterial toxins often have a crucial function in the pathogenesis of infectious diseases.

Considering the burden of infectious diseases, it is essential to identify and characterize virulent proteins from pathogenic bacteria, as these are indispensable for the pathogen's survival. Due to their significance, several databases have been developed for virulent proteins, such as the MVirDb (Zhou et al., 2006) and Virulence Factor Database (VFDB) (Chen et al., 2005; Liu et al., 2018; Liu et al., 2021). However, the experimental approaches to identifying such proteins are time‐consuming and expensive. Furthermore, experimental determination of a gene's involvement in disease requires knockout studies (Paul et al., 2021) or mutations in the putative virulence genes, which require a lot of experimental resources and time, therefore, a costly endeavor. Hence, there is a requirement for faster methods to determine the most putative virulent proteins quickly and efficiently before experimental assays are carried out.

Computational approaches are valuable for predicting virulent proteins based on sequence data. The advent of genomics and sequencing technologies has enabled the rapid identification of such proteins (Burrack & Higgins, 2007) and the development of computational methods for this purpose. Many of these computational methods rely on sequence similarity, motif, and domain search; however, these methods have limited efficiency in cases where the sequences share little to no sequence similarity. Hence, several sequence‐independent methods for predicting such proteins are based on machine learning (Bonetta & Valentino, 2020), such as support vector machines (SVM), SPAAN (Sachdeva et al., 2004), VirulentPred (Garg & Gupta, 2008), VICMpred (Saha & Raghava, 2006), MP3 (Gupta et al., 2014), and others.

In one of our past studies, we developed VirulentPred, an SVM‐based method for identifying virulent proteins in bacteria (Garg & Gupta, 2008). Here, we present an advanced and improved version of our previously developed tool, VirulentPred 2.0, with a standalone version and a web‐based GUI interface to facilitate rapid identification of these proteins. For VirulentPred 2.0, we used only experimentally verified virulent proteins to develop the prediction model and achieved an accuracy of 85.18%. This makes our study stand out from others using putative or hypothetical proteins, apart from experimentally validated virulent proteins, to train the models. Our method will have profound implications for novel drug targets and vaccine candidate predictions.

## 2 MATERIALS AND METHODS

### 2.1 Data source

**FIGURE 1:**
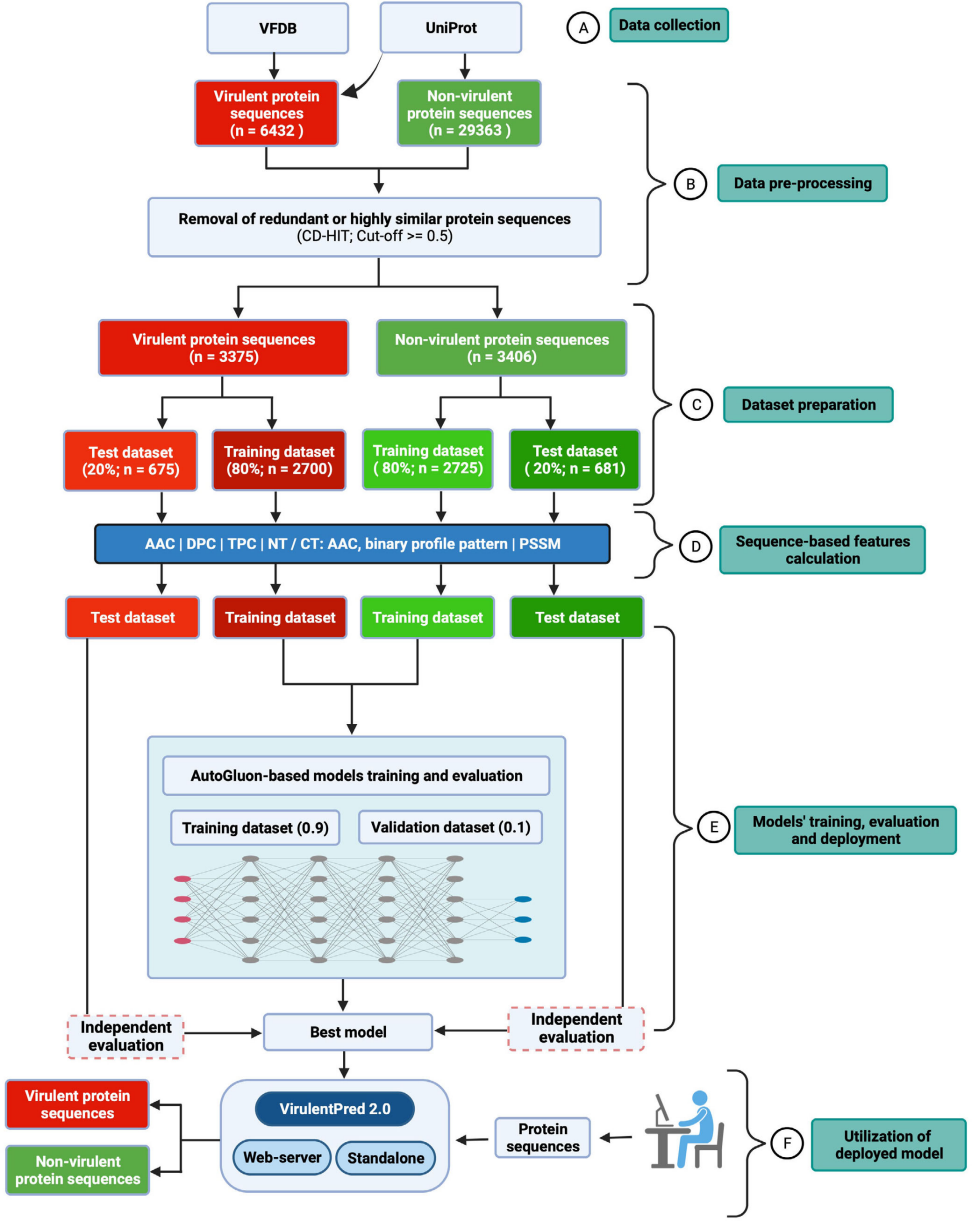
A flowchart depicting the overall approach implemented for the data collection, pre‐processing, model building and deployment of the best model for VirulentPred 2.0.

The generation of the current positive data set began by retrieving 3580 and 2852 virulent protein sequences from the VFDB (Liu et al., 2021) and UniProt (UniProt, 2021) databases, respectively (Figure 1).

### 2.2 Data pre‐processing

The core dataset file was downloaded from VFDB, whereas we used virulence‐related keywords such as virulence, adhesin, adhesions, toxin, invasion, capsule and so forth to obtain sequences from UniProt (UniProt, 2021). Further, these 6432 sequences were strictly screened to filter out the entries labeled as “probable,” putative, similarity, fragments, hypothetical, unknown, and possible (Figure 1).

The annotated sequences of bacterial enzymes were downloaded from the UniProt (UniProt, 2021) database to make a negative dataset. Here, the nonvirulent protein sequences were searched mainly in the bacterial proteomes from which virulent protein sequences were obtained for the positive dataset. This dataset was screened strictly to generate a high‐quality negative set.

Subsequently, the sequences were clustered and compared using CD‐HIT (Fu et al., 2012) at a cut‐off value of 0.5 to remove identical sequences and reduce redundancy among positive and negative datasets. First, we obtained 3403 sequences out of 6432 using CD‐HIT for the positive dataset. Further, to remove related lines among positive and negative sets, these datasets were combined and CD‐HIT was reapplied with a cut‐off value of 0.5, resulting in 3375 virulent protein sequences. Eventually, the processed dataset consisted of nonredundant 6781 sequences, comprising 3375 virulent and 3406 nonvirulent protein sequences (randomly selected to balance the positive dataset) to generate trained models (Figure 1). The distribution of virulent protein sequences from different bacterial pathogens after refinement is shown in Table S1.

### 2.3 Preparation of training and test datasets

Preparation of training and test datasets is for machine learning. In the present study, the positive and negative datasets were randomly shuffled and divided into 80% training and 20% test datasets. To ensure the absence of homologous proteins between these training and test datasets, we built a local database with training dataset sequences (both positive and negative dataset protein sequences, *n* = 5425), and performed a PSI‐BLAST search (using PSI‐BLAST run options “‐e 0.001 ‐j 3 ‐m 9 ‐v 1 ‐b 1”, where e is E‐value, j is the maximum number of passes, and the rest of the variables are related to PSI‐BLAST output format) against this database with the test datasets protein sequences (*n* = 1356). From Table S2, only 6.05% (*n* = 82) of test dataset protein sequences showed similarity with training dataset proteins at an identity percentage value of ≥50. Table 1 provides the distribution of positive and negative dataset protein sequences used in the present study. Further, the training dataset was randomly divided into an actual training set (0.9 fraction of the data) and validation set (holdout 0.1 fraction of data) through an in‐built feature of AutoGluon (Erickson et al., 2020), for the training and internal evaluation of machine learning (ML) models, respectively.

**TABLE 1:** Distribution of protein sequences in the training and test datasets.

Dataset type	Total number of protein sequences	Number of protein sequences in training dataset (80%)	Number of protein sequences in test dataset (20%)
Positive dataset	3375	2700	675
Negative dataset	3406	2725	681
Both datasets	6781	5425	1356

### 2.4 Calculation of protein sequence composition

The standalone package named GPSR (Saini et al., n.d.) was used for the calculation of the amino acid composition (AAC), dipeptide composition (DPC), and tripeptide composition (TPC) of virulent and nonvirulent protein sequences.

### 2.5 Calculation of protein sequences PSSM profiles

We used an in‐house PERL script to calculate the PSI‐BLAST (Altschul et al., 1997) generated PSSM profiles. A PSI‐BLAST iterative search was performed against the SwissProt database with an *E*‐value cut‐off 0.001 for the calculations.

### 2.6 Techniques used for the training of ML models

We used 14 different ML algorithms available in the AutoGluon package (Erickson et al., 2020) for the training and performance evaluation of ML models (Table 2).

**TABLE 2:** List of algorithms used for the training and evaluation of VirulentPred 2.0 ML models.

Sr. No.	Algorithm name	Reference
1	CatBoost	https://catboost.ai/
2	ExtraTreesEntr	https://scikit‐learn.org/stable/modules/generated/sklearn.ensemble.ExtraTreesClassifier.html#sklearn.ensemble.ExtraTreesClassifier
3	ExtraTreesGini	https://scikit‐learn.org/stable/modules/generated/sklearn.ensemble.ExtraTreesClassifier.html#sklearn.ensemble.ExtraTreesClassifier
4	KNeighborsDist	https://scikit‐learn.org/stable/modules/generated/sklearn.neighbors.KNeighborsClassifier.html
5	KNeighborsUnif	https://scikit‐learn.org/stable/modules/generated/sklearn.neighbors.KNeighborsClassifier.html
6	LightGBM	https://lightgbm.readthedocs.io/en/latest/
7	LightGBMLarge	https://lightgbm.readthedocs.io/en/latest/
8	LightGBMXT	https://lightgbm.readthedocs.io/en/latest/
9	NeuralNetFastAI	https://auto.gluon.ai/0.4.0/api/autogluon.tabular.models.html
10	NeuralNetTorch	https://auto.gluon.ai/0.4.0/api/autogluon.tabular.models.html
11	RandomForestEntr	https://scikit‐learn.org/stable/modules/generated/sklearn.ensemble.RandomForestClassifier.html
12	RandomForestGini	https://scikit‐learn.org/stable/modules/generated/sklearn.ensemble.RandomForestClassifier.html
13	WeightedEnsemble	https://www.cs.cornell.edu/~alexn/papers/shotgun.icml04.revised.rev2.pdf
14	XGBoost	https://xgboost.readthedocs.io/en/latest/

### 2.7 PSI‐BLAST‐based classification of proteins

We used two approaches to evaluate PSI‐BLAST's performance for classifying virulent and nonvirulent proteins. Firstly, a local database of training dataset protein sequences (*n* = 5425) was generated, and a sequence similarity search of test dataset sequences (*n* = 1356), against this database was performed through PSI‐BLAST (using the same PSI‐BLAST run parameters as mentioned in the Section 2.3). Secondly, the PSI‐BLAST search performance (using the same PSI‐BLAST run parameters as mentioned in the Section 2.3) was also evaluated through a five‐fold cross‐validation technique applied to the training datasets protein sequences. For a five‐fold cross‐validation‐based PSI‐BLAST evaluation, the virulent and nonvirulent protein sequences training dataset was shuffled and divided into five equally sized parts. The PSI‐BLAST search was performed five times so that each time, one part was used as a test set, and the remaining four parts were used to build a local database against which the search was performed. Thus, each part was used at least once for training and testing.

### 2.8 Criteria used for the selection of best ML models

The training dataset is used for training the machine learning models (0.9 fraction) and identifying the best‐performing models with the help of the validation dataset (holdout 0.1 fraction from the training dataset). The models with the highest accuracy on the validation dataset are automatically opted for and saved as the best models by AutoGluon (Erickson et al., 2020). The saved models are further evaluated with a test dataset to estimate their real‐life performance. The best‐performing models on validation and test datasets are deployed on the VirulentPred 2.0 web server. Moreover, the best model (PSSM‐based) is also available on the VirulentPred 2.0 web server for their usage as online predictors by users on their desktops or workstations.

## 3 RESULTS

To develop the AutoGluon‐based ML models, several input features, such as AAC of whole protein sequences, N/C‐terminus residues AAC and binary profile patterns (BPPs), PSSM profiles, and so forth, were used. The performances of ML models with these input features are given in the subsequent sections.

### 3.1 Whole AAC‐based models

Firstly, the best model trained with the whole AAC of protein sequences achieved the highest accuracy of 80.85% and 74.85% for validation and test datasets, respectively (Table 3). For DPC‐based models, the highest accuracy of 79.37% and 74.56% was achieved for validation and test datasets, respectively. Similarly, 74.40% and 69.91% accuracy were achieved for TPC‐based models for validation and test datasets, respectively. Thus, all the AAC‐based models performed with moderate accuracy.

**TABLE 3:** Performances of models trained with the whole amino acid composition of protein sequences.

Data description		Validation dataset performance	Test dataset performance			
Input data	Number of input vectors	Accuracy	Sensitivity	Specificity	Accuracy	MCC
Whole amino acid composition	20	80.85	73.48	76.21	74.85	0.50
Dipeptide composition	400	79.37	71.41	77.68	74.56	0.49
Tripeptide composition	8000	74.40	72.00	67.84	69.91	0.40

Abbreviation: MCC, Mathew's correlation coefficient.

### 3.2 N and C‐terminus residues' AAC‐based models

The AAC of N and C‐terminus protein/peptide sequences are essential features in predicting various biological properties, for example, antimicrobial, antibiofilm, cell‐penetrating, tumor homing, and various other activities (Cooper & Marsden, 2017; Gautam et al., 2013; Krishna & Englander, 2005; Lata et al., 2010; Petsalaki et al., 2006; Sharma et al., 2013, 2016; Yamada et al., 2017). However, in the present study, the highest accuracy of 59.51% (with test dataset) was achieved with the AAC of the C‐terminal first 10 residues (CT10) as input features to the machine learning models (Table 4).

**TABLE 4:** Performances of models trained with the N and C‐terminus residues composition of protein sequences.

Data description		Validation dataset performance	Test dataset performance			
Input data	Number of input vectors	Accuracy	Sensitivity	Specificity	Accuracy	MCC
NT5	20	62.62	52.89	60.06	56.49	0.13
CT5	20	60.22	49.48	59.77	54.65	0.09
NT10	20	58.75	52.59	62.56	57.60	0.15
CT10	20	64.46	53.48	65.49	59.51	0.19
NTCT5	20	62.25	46.67	64.32	55.53	0.11
NTCT10	20	63.72	50.07	67.69	58.92	0.18

### 3.3 N and C‐terminus residues' binary profile pattern‐based models

Specific residues at the N and/or C‐terminus of the sequences (in a position‐specific manner) have been reported to be associated with the biological activities of proteins and peptides (Sharma et al., 2013, 2016). Therefore, in the present study, an attempt has been made to predict the virulent protein sequences with the help of N and C‐terminus residues' BPPs. As evident from Table 5, the highest accuracy of 57.51% (test dataset) was achieved with the BPPs of the N‐terminal first 10 residues (NT10) as input features to the machine learning models.

**TABLE 5:** Performances of models trained with the N and C‐terminus residues' binary profile patterns.

Data description		Validation set performance	Test dataset performance			
Input data	Number of input vectors	Accuracy	Sensitivity	Specificity	Accuracy	MCC
NT5	100	62.25	52.89	58.59	55.75	0.11
CT5	100	62.62	52.44	56.39	54.42	0.09
NT10	100	61.88	52.59	61.67	57.15	0.14
CT10	100	58.56	53.63	60.06	56.86	0.14
NTCT5	200	61.14	55.41	57.86	56.64	0.13
NTCT10	400	65.19	47.85	64.90	56.42	0.13

### 3.4 Performance of hybrid models

Eleven hybrid ML models were developed with all possible combinations of the calculated input features (Table 6). The highest test dataset accuracy of 84.51% was achieved with a hybrid of AAC, DPC, and PSSM as input features to train the ML model.

**TABLE 6:** Performances of hybrid models trained with the whole amino acid composition and/or PSSM profiles of protein sequences.

Data description		Validation dataset performance	Test dataset performance			
Input data	Number of input vectors	Accuracy	Sensitivity	Specificity	Accuracy	MCC
AAC + DPC	420	84.35	72.00	78.12	75.07	0.50
DPC + PSSM	800	84.35	86.22	80.76	83.48	0.67
AAC + PSSM	420	88.95	84.44	82.23	83.33	0.67
TPC + PSSM	8400	85.27	82.96	83.99	83.48	0.67
AAC + TPC	8020	78.82	71.70	79.15	75.44	0.51
DPC + TPC	8400	76.43	72.89	77.53	75.22	0.50
AAC + DPC + TPC	8420	81.03	73.93	77.24	75.59	0.51
AAC + DPC + PSSM	820	86.56	85.78	83.26	84.51	0.69
DPC + TPC + PSSM	8800	86.19	77.63	88.11	82.89	0.66
AAC + TPC + PSSM	8420	82.14	86.22	79.00	82.60	0.65
AAC + DPC + TPC + PSSM	8820	85.27	82.81	83.99	83.41	0.67

Abbreviations: AAC, amino acid composition; DPC, dipeptide composition; TPC, tripeptide composition.

### 3.5 PSI‐BLAST‐based classification of virulent and nonvirulent proteins

Retrospectively, while working on the VirulentPred server (Garg & Gupta, 2008), we have seen that PSI‐BLAST (Altschul et al., 1997) alone cannot efficiently classify virulent and nonvirulent protein sequences. Using PSI‐BLAST as a similarity‐based search model, we could predict 52.5% and 51.7% of virulent and nonvirulent proteins, respectively, leading to an overall accuracy of 52.1%. Therefore, AI methods always have the edge over conventional similarity‐based search methods.

A two‐step process was used to check the same for the present study. A PSI‐BLAST‐based search was performed with test dataset proteins in the first step. Thus, search hits were retrieved for only 71.46% of test dataset protein sequences (*n* = 969) when no identity (%) threshold was applied (Table S3). During the search, if a test dataset protein sequence showed identity with more than one training dataset of protein sequences, the functional class of the highest identical subject protein (with the highest identity % value) was assigned as the predicted functional class (virulent or nonvirulent) to the query test protein. Thus, PSI‐BLAST failed to detect hits for 28.54% of test dataset proteins (*n* = 387) at no identity (%) threshold. Although higher accuracy was achieved with BLAST, but with a fewer protein sequences than the ML‐based method. However, for a rational sequence identity/similarity‐based functional class assignment, an identity threshold of at least ≥30% is used. At this identity threshold of ≥30% between query and subject proteins, BLAST could annotate just 55.53% of test dataset proteins. Thus, it is clear from this analysis that BLAST failed to detect 44.47% of test dataset protein sequences even when evaluated with a small and well‐curated dataset of annotated virulent and nonvirulent proteins. Moreover, at the highest identity thresholds of ≥50, hits were retrieved for only 6.05% of test dataset protein sequences.

In the second step, only 68.85% of protein sequences retrieved hits showed similarity with the local sequences database through a five‐fold cross‐validation technique when no identity cut‐off was applied (Table S4). Functional classes were assigned to query test dataset sequences following the rules used in the first step. At an identity cut‐off of ≥30%, only 52.70% of protein sequences retrieved hits. At the highest identity thresholds of ≥50%, the hits were retrieved for only 5.33% of protein sequences. Thus, it is clear from these two evaluations that PSI‐BLAST is not sufficient to identify the virulent proteins.

### 3.6 Comparative performance of VirulentPred models

The comparative evaluation with the latest test dataset (from VirulentPred 2.0) helped assess the performance improvement achieved by VirulentPred 2.0 over the previously developed VirulentPred. Table 7 provides the comparative performance of the best models from VirulentPred 2.0 and VirulentPred 1.0. It can be seen that VirulentPred 2.0 is more accurate than its previous version. For example, in the case of the “Cascade SVM classifier”, an approximately 7% increase in the prediction accuracy (from 75.74% to 82.82%) is achieved with VirulentPred 2.0. Whereas, for PSSM profile‐based models, about an 11% increase in the prediction accuracy (from 74.19% to 85.18%) is achieved with VirulentPred 2.0. In the case of threshold independent performance evaluation, a significant improvement in the AUC value, that is, from 0.85 (reported in the previous version of VirulentPred, for the PSSM‐profiles‐based model) to 0.924 is achieved (Figure S1). Therefore, the PSSM profile‐based model is deployed as the best predictor model in VirulentPred 2.0.

**TABLE 7:** Performance of best models from VirulentPred 2.0 and VirulentPred with latest test dataset.

Data description	Validation dataset performance	Test dataset performance			
Model type	Accuracy	Sensitivity	Specificity	Accuracy	MCC
VirulentPred 2.0 (Cascade classifier‐based model)	100	79.11	86.49	82.82	0.66
VirulentPred (Cascade SVM‐based classifier model)	N/A	77.48	74.01	75.74	0.52
VirulentPred 2.0 (PSSM profile‐based model)	84.71	85.33	85.02	85.18	0.70
VirulentPred (PSSM profile‐based model)	N/A	68.44	79.88	74.19	0.49

Abbreviation: SVM, support vector machines.

### 3.7 Advantages of VirulentPred 2.0 standalone over web‐server

VirulentPred 2.0 web‐server version can be used by biologists who wish to make predictions for a few sequences or those who do not have any knowledge of software installation. The standalone version can be installed on local desktops/workstations for bulk classifications. Furthermore, depending on the availability of hardware configuration (at the user's end), multiple runs can be applied simultaneously by supplying multiple input sequences files (after splitting thousands or lakhs of sequences containing big files into smaller hundreds of sequences containing files). Moreover, long waiting queues on the web server can be avoided using the standalone VirulentPred 2.0.

## 4 DISCUSSION

The bacterial virulence factors, such as virulent proteins, carbohydrates, etc., are essential for bacterial survival and proliferation within the host cells. A major portion of virulent factors is constituted of virulent proteins. The latter plays an essential role in bacterial attachment, entry, movement, and survival into the host cells. For pan‐bacterial metagenomic analyses, a group has organized the virulence factors into 14 general categories (Liu et al., 2021).

Identifying the virulent proteins synthesized by clinically important pathogenic bacteria is an important research task in infectious disease biology. However, due to the advancements in sequencing technologies, genomics and proteomics, data are being generated rapidly, which makes the experimental identification of these proteins challenging and time‐consuming. Computational identification of virulent proteins can facilitate experimental identification of virulent proteins. In 2008, we developed a computational method named “VirulentPred” to classify bacterial virulent and nonvirulent proteins (Garg & Gupta, 2008). VirulentPred 1.0 is widely used by researchers across the globe for the identification of virulent bacterial proteins. However, the real‐life success of a machine learning‐based method depends on its regular, improved training and evaluation with the latest available datasets. The availability of novel virulent and nonvirulent protein sequences in the published literature motivated us to develop a new and highly accurate version “VirulentPred 2.0.” For developing VirulentPred 2.0, we used virulent proteins from 32 genera of pathogenic bacteria in this study, whereas in the previous version of VirulentPred, we used protein sequences from only 12 bacterial genera. Moreover, the new version is trained and evaluated with the contemporary ML techniques implemented through the AutoGluon package. The best models developed in the present study are WeightedEnsemble_L2 types.

Like the previous version, the algorithms evaluated by us did not perform well with the AAC, DPC, and TPC of the protein sequences. Moreover, N and C‐terminus datasets AAC and BPPs failed to achieve satisfactory classification accuracy. In the case of the hybrid approach, a hybrid of AAC, DPC, and PSSM‐based ML model performed with the highest accuracy. However, the VirulentPred 2.0 algorithms trained and evaluated with PSSM and cascade classifier (AAC, DPC, TPC, and PSSM) of protein sequences outperformed the previously developed models in the external or independent validation with the latest test dataset (Table 7).

A significant improvement in the classification accuracy is achieved after the development of VirulentPred 2.0 models. The in‐built facility of AutoGluon, that is, weighted ensemble layers and the more extensive and updated training dataset of virulent and nonvirulent proteins, have increased classification accuracy. In contrast to the older SVM cascade model, the present best model is trained and evaluated with the PSSM profiles of the protein sequences. This indicates the importance of evolutionary information of proteins towards their classification into virulent and nonvirulent types.

## 5 CONCLUSION

A new version of the VirulentPred has been developed for classifying virulent and nonvirulent proteins with higher efficiency. The new user‐friendly standalone version of VirulentPred 2.0 using local computers can classify a few protein sequences to whole bacterial proteomes in a high‐throughput mode. In future, we will develop VirulentPred further to include functions like nucleotide sequence‐based predictions.

## AVAILABILITY

The VirulentPred 2.0 web server and standalone version are available at https://bioinfo.icgeb.res.in/virulent2/.

## CODES AVAILABILITY

All the materials used in the present study are available online at https://bioinfo.icgeb.res.in/virulent2/. The VirulentPred 2.0 codes and the best model (PSSM‐based) for prediction through a standalone version are freely available at https://bioinfo.icgeb.res.in/virulent2/down.html.

## AUTHOR CONTRIBUTIONS

Dinesh Gupta, Aarti Garg, and Arun Sharma conceptualized the study. Aarti Garg curated the datasets required for the study. Jayashree Ramana calculated the PSSM profiles from the protein sequences. Arun Sharma carried out the machine learning studies and developed web server and standalone versions of VirulentPred 2.0. Arun Sharma, Aarti Garg, Jayashree Ramana, and Dinesh Gupta prepared the manuscript. All authors reviewed and approved the final version of the manuscript.

## FUNDING INFORMATION

The present work was conducted in a Bioinformatics facility, supported by the Department of Biotechnology, India (grant ID: BT/PR40151/BTIS/137/5/2021.

## CONFLICT OF INTEREST STATEMENT

The authors declare that the research was conducted in the absence of any commercial or financial relationships that could be construed as a potential conflict of interest.

## Supporting information


**Data S1.** Supporting information.

## Data Availability

The test datasets used for the independent evaluation of prediction models may be obtained from https://bioinfo.icgeb.res.in/virulent2/.
