# Supplementary material for: VirulentPred 2.0: An improved method for prediction of virulent proteins in bacterial pathogens
Source: Protein Sci. 2023 Dec 1;32(12):e4808. doi: 10.1002/pro.4808 (PMC10659933; doi:10.1002/pro.4808)
Supplement: Supplementary file 1 — Data S1. Supporting information. [file PRO-32-e4808-s001.docx]

**Supplementary Information**

**VirulentPred 2.0: an improved method for prediction of virulent proteins in bacterial pathogens**

Arun Sharma^#^, Aarti Garg^#^, Jayashree Ramana, Dinesh Gupta**^*^**

Translational Bioinformatics Group, International Centre for Genetic Engineering and Biotechnology (ICGEB), Aruna Asaf Ali Marg, New Delhi-110067, India.

***Address for correspondence:**

Dr. Dinesh Gupta

Group Leader,

Translational Bioinformatics Group,

International Centre for Genetic Engineering and Biotechnology (ICGEB),

New Delhi, India-110067.

Phone number: +91-11-26742184

Mail address: [dinesh@icgeb.res.in](mailto:dinesh@icgeb.res.in)

**^#^**Equal contributors

**Table S1. The distribution of virulent protein sequences from different bacterial pathogens.**

| **Sr.No.** | **Bacteria genus** | **No. of protein sequences** |
| --- | --- | --- |
| 1 | Acinetobacter | 42 |
| 2 | Aeromonas | 181 |
| 3 | Anaplasma | 21 |
| 4 | Bacillus | 92 |
| 5 | Bartonella | 64 |
| 6 | Bordetella | 62 |
| 7 | Brucella | 55 |
| 8 | Burkholderia | 143 |
| 9 | Campylobacter | 117 |
| 10 | Chlamydia | 52 |
| 11 | Clostridium | 41 |
| 12 | Corynebacterium | 16 |
| 13 | Coxiella | 147 |
| 14 | Enterococcus | 39 |
| 15 | Escherichia | 235 |
| 16 | Francisella | 99 |
| 17 | Haemophilus | 69 |
| 18 | Helicobacter | 101 |
| 19 | Klebsiella | 89 |
| 20 | Legionella | 383 |
| 21 | Listeria | 44 |
| 22 | Mycobacterium | 172 |
| 23 | Mycoplasma | 26 |
| 24 | Neisseria | 53 |
| 25 | Pseudomonas | 235 |
| 26 | Rickettsia | 28 |
| 27 | Salmonella | 156 |
| 28 | Shigella | 74 |
| 29 | Staphylococcus | 140 |
| 30 | Streptococcus | 121 |
| 31 | Vibrio | 165 |
| 32 | Yersinia | 113 |

**Table S2. Results of PSI-BLAST-based identity percentage check.** The table shows number of test dataset proteins (n=1356) for which hits found and not found during PSI-BLAST-based similarity check against the local database of training dataset proteins (n=5425) at different identity (%) thresholds.

| **Sr.No.** | **Identity (%)** | **Hits found (%)** | **No hits found (%)** |
| --- | --- | --- | --- |
| 1 | None | 969 (71.46) | 387 (28.54) |
| 2 | ≥20% | 968 (71.39) | 388 (28.61) |
| 3 | ≥30% | 753 (55.53) | 603 (44.47) |
| 4 | ≥40% | 443 (32.67) | 913 (67.33) |
| 5 | ≥50% | 82 (6.05) | 1274 (93.95) |

**Table S3. PSI-BLAST-based test dataset search results against the local database of training dataset sequences at different identity (%) values.**

| Sr.No. | Accuracy | Sensitivity | Specificity | MCC | Identity (%) | Hits found (%) | No hits found (%) |
| --- | --- | --- | --- | --- | --- | --- | --- |
| 1 | 89.16 | 90.4 | 87.96 | 0.78 | None | 969 (71.46) | 387 (28.54) |
| 2 | 89.26 | 90.4 | 88.14 | 0.79 | ≥20% | 968 (71.39) | 388 (28.61) |
| 3 | 90.84 | 92.69 | 89.29 | 0.82 | ≥30% | 753 (55.53) | 603 (44.47) |
| 4 | 91.2 | 91.67 | 90.91 | 0.82 | ≥40% | 443 (32.67) | 913 (67.33) |
| 5 | 96.34 | 100 | 93.75 | 0.93 | ≥50% | 82  (6.05) | 1274 (93.95) |

**Table S4. PSI-BLAST-based five-fold cross validation of training dataset protein sequences at different identity (%) values.**

| Sr.No. | Accuracy | Sensitivity | Specificity | MCC | Identity (%) | Hits found (%) | No hits found (%) |
| --- | --- | --- | --- | --- | --- | --- | --- |
| 1 | 89.18 | 88.09 | 90.21 | 0.78 | None | 3735 (68.85) | 1690 (31.15) |
| 2 | 89.21 | 88.15 | 90.21 | 0.78 | ≥20% | 3726 (68.68) | 1699 (31.32) |
| 3 | 90.98 | 88.96 | 92.56 | 0.82 | ≥30% | 2859 (52.70) | 2566 (47.30) |
| 4 | 92.93 | 89.46 | 94.88 | 0.85 | ≥40% | 1556 (28.68) | 3869 (71.32) |
| 5 | 94.46 | 94.12 | 94.71 | 0.89 | ≥50% | 289  (5.33) | 5136 (94.67) |

**Figure S1. ROC curve for the PSSM profile-based model (evaluated with latest test dataset (n=1356)) which is deployed on VirulentPred 2.0 web server and standalone application.**

**
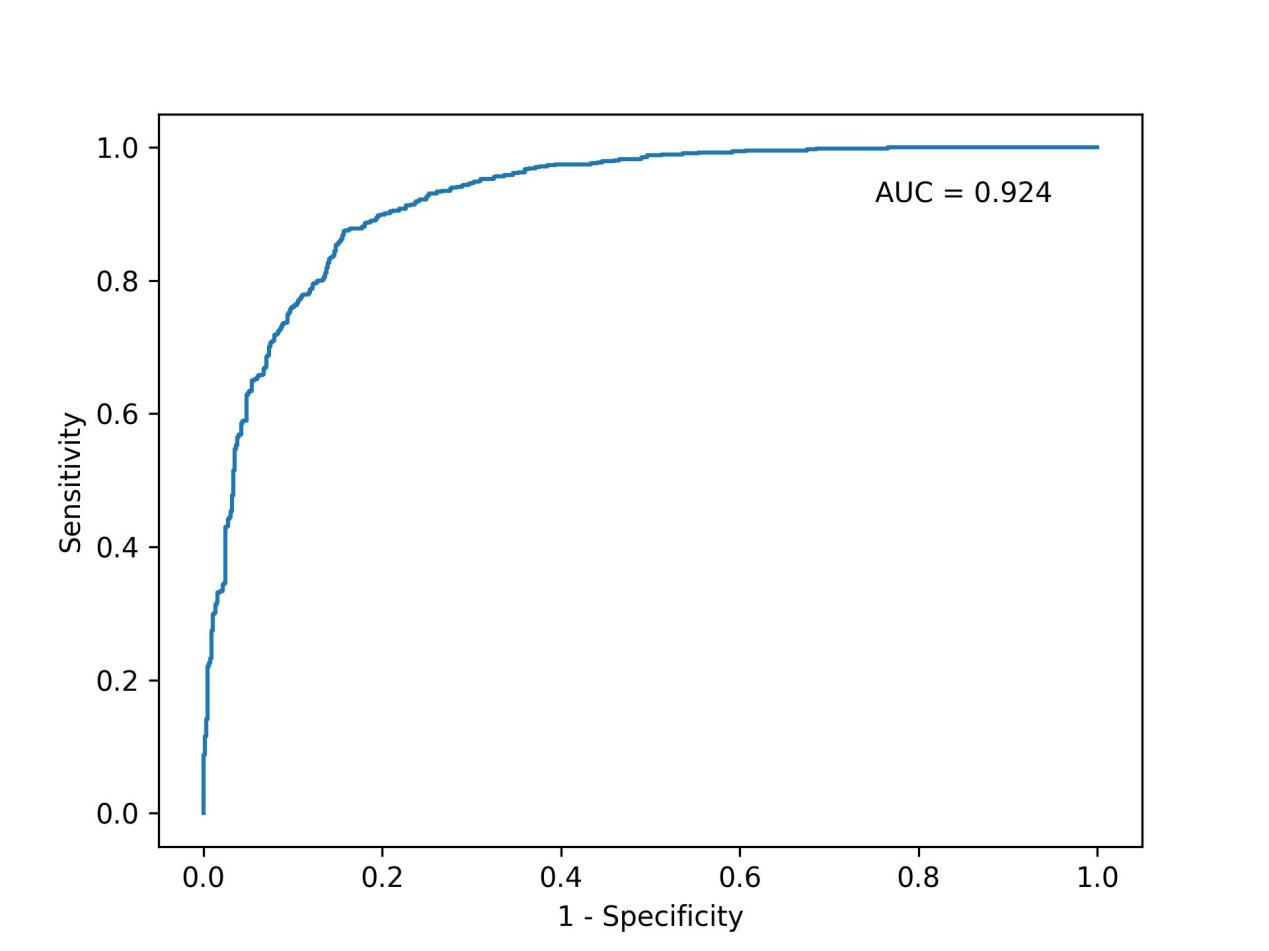
**
